# SOX9 Expression Is Increased in Alzheimer’s Disease (AD) and Is Associated With Disease Progression and APOE4 Genotype: A Computational Approach

**DOI:** 10.7759/cureus.36129

**Published:** 2023-03-14

**Authors:** Aliaa A Alamoudi

**Affiliations:** 1 Clinical Biochemistry, Faculty of Medicine, King Abdulaziz University, Jeddah, SAU; 2 Regenerative Medicine Unit, King Fahad Medical Research Center, Jeddah, SAU

**Keywords:** oxidative phosphorylation, apoe, braak, sox9, alzheimer

## Abstract

Introduction: Alzheimer’s disease (AD) is a neurodegenerative disease characterized by depositions of amyloid-β protein leading to neuronal loss. Despite our understanding of the disease several gaps remain, including the role of astrocytes and astrocytic genes in the disease development and progression. Recently, some reports have suggested that SOX9 transcription factor (TF), an important mediator of astrocyte differentiation and maturation, might be linked to AD. Using human AD publicly available dataset, we aimed to analyze SOX9 expression and its relation to disease.

Methodology: The AD gene expression data set was obtained from National Center for Bioinformatics-Gene Expression Omnibus (NCBI-GEO). The GSE48350 consisted of mRNA microarray data from 55 normal controls (173 samples) and 26 AD cases (81 samples) obtained, from four brain regions. The SOX9 expression profile and correlations were analyzed using the R2 Genomics Analysis and Visualization platform.

Results: The SOX9 was significantly upregulated (p<0.001) in AD tissue compared to control cases. The increased expression appeared to be more in the entorhinal cortex (EC) and hippocampus (HC) regions. The SOX9 expression positively correlated with BRAAK stages (p<0.05). Interestingly in AD patients the SOX9 expression was significantly less in APOE3/3 genotypes compared with genotypes containing APOE4 allele. The SOX9 expression negatively correlated with oxidative phosphorylation genes which could suggest a metabolic role for the TF.

Conclusion: From these data we hypothesize that SOX9 acts as a metabolic regulator responding to lipid metabolism disruption associated with APOE4 genotypes. In turn, SOX9 expression could be associated with astrocyte maturation and survival in the disease contributing thus to disease burden and disease progression.

## Introduction

Alzheimer’s disease (AD) is a neurodegenerative disease that is considered the most common cause of dementia. It is characterized by extracellular amyloid plaques, which are deposits of amyloid-β (Aβ) protein, in addition to intracellular neurofibrillary tangles due to abnormal tau hyperphosphorylation [1-2]. It is believed that the extensive deposition of the Aβ is responsible for triggering the intracellular tau modifications. Ultimately, these changes result in severe synaptic and neuronal loss which results in dementia seen with AD.

Pathological staging of AD is the gold standard diagnostics in postmortem cases and was proposed by Braak [3-4]. Stages consisted of the following regions: Braak I (transentorhinal), Braak II (entorhinal and hippocampus, HC), Braak III (amygdala, parahippocampal gyrus, fusiform gyrus, and lingual gyrus), Braak IV (insula, inferior temporal, lateral temporal, posterior cingulate, and inferior parietal), Braak V (orbitofrontal, superior temporal, inferior frontal, cuneus, anterior cingulate, supramarginal gyrus, lateral occipital, precuneus, superior parietal, superior frontal, and rostromedial frontal), and Braak VI (paracentral, postcentral, precentral, and pericalcarine) [3-4].

Although a small subset of patients, known as the early onset familial AD, inherit the diseases in an autosomal dominant manner, the majority of AD cases are late onset and are not inherited [5]. Multi factors including genetics, environmental, and lifestyle are believed to play a role in the disease. Of great interest is APOE polymorphism, which is described as the most important genetic risk factor for the late onset [5]. In the brain APOE protein is produced mainly by astrocytes and activated microglia. Heterozygote and homozygotes carriers of the APOE4 allele have an increased 3-4 and 12-15 times risk of dementia respectively compared with APOE ε3 carriers. It is believed that APOE4 isoform is associated with a slower clearance of Aβ protein.

It is known that astrocytes are one of the most numerous glial cells in the central nervous system (CNS) which play several vital functions [2]. They play a role in regulating blood flow and the blood brain barrier, synapse formation and remodeling, extracellular fluid and ion homeostasis, the release and up-take of neurotransmitters, in addition to a role in regulating energy metabolism. It is, therefore, not surprising that disruption of these homeostatic functions in astrocytes have been linked to the onset and progression of various neurogenerative diseases. Indeed, through different mechanisms, astrocytes have been linked to the pathophysiology and progression of AD [2]. In addition, AD astrocytes were found to have a different gene expression profile than normal cells, which also differed between acute and chronic conditions.

The SRY-box transcription factor 9 (SOX9) belongs to the SOX family which during embryogenesis is involved in cell fate, regulating differentiation processes [6]. The transcription factor (TF) plays a major role in chondrocyte differentiation, however, it is also very highly expressed in astrocyte and neuronal progenitor cells, and appears to play a role in gliogenesis and astrocyte differentiation [7]. The TF was found to be upregulated in diseases such as multiple strokes and middle cerebral artery occlusion (MCAO) [8]. Recent animal studies have suggested that upregulation of SOX9 could promote ischemic brain injury (IBI) mainly through upregulating FOXO3 which itself is an important regulator of various cellular mechanisms including apoptosis, and autophagy metabolism [9]. Currently very limited studies have studied SOX9 association with AD, however, interestingly a recent study has shown that miR-22-3p can have a protective effect in AD by acting on SOX9 in the HC [10].

It is known that computational methods have led to identifying tissue-based molecular biomarkers through analyzing gene expression data of different disease datasets. Not only can this lead to better understanding of the pathophysiology and molecular aspects of the disease but can also lead to identifying biomarkers and expression signatures that can monitor disease progression and identify drug targets. Using secondary human dataset we aimed to study the association of SOX9 expression with AD by employing robust bioinformatic gene network analysis and advanced statistical tools.

## Materials and methods

Datasets collection for AD

The AD gene expression dataset was obtained from National Center for Bioinformatics-Gene Expression Omnibus (NCBI-GEO). The GSE48350 consisted of mRNA microarray data from 55 normal controls (aged 20-99 years) and 26 AD cases (aged 74-99 years) obtained, from four brain regions: entorhinal cortex (EC) HC, post-central gyrus (PCG), and superior frontal gyrus (SFG). Samples were postmortem autopsy samples obtained from seven well-established American institutes of Aging Alzheimer’s disease brain banks. A total of 173 normal samples and 81 AD samples were used to generate microarray data using Affymetrix HgU133 plus 2.0 arrays.

SOX9 expression

To determine differentially expressed genes (DEGs), a differential expression analysis was carried out using both NCBI webtool GEO2R and R2 Genomics Analysis and Visualization platform [11-13]. And adjusted p value <0.01 was used as a cutoff. To assess the expression of SOX9 in AD vs. control, in various BRAAK stages and in APOE genotypes R2 Genomics Analysis and Visualization platform was used. R2 platform was used to conduct statistical analysis using t-test or ANOVA when comparing two groups or more than two groups respectively. p<0.05 was considered significant. All figures were generated using R2 platform. Data were presented as median and inter quartile range (IQR) for better presentation of skewed data, especially with small number of samples and with outliers.

SOX9 correlation with oxidative phosphorylation genes

An adjusted false discovery rate (FDR) with a cut off p-value of 0.05 was used for correlation analysis. Gene ontology was used to find metabolic pathways of biological processes which significantly correlated with SOX9 in the data set.

## Results

SOX9 is significantly upregulated in AD brain tissue

**Figure 1:**
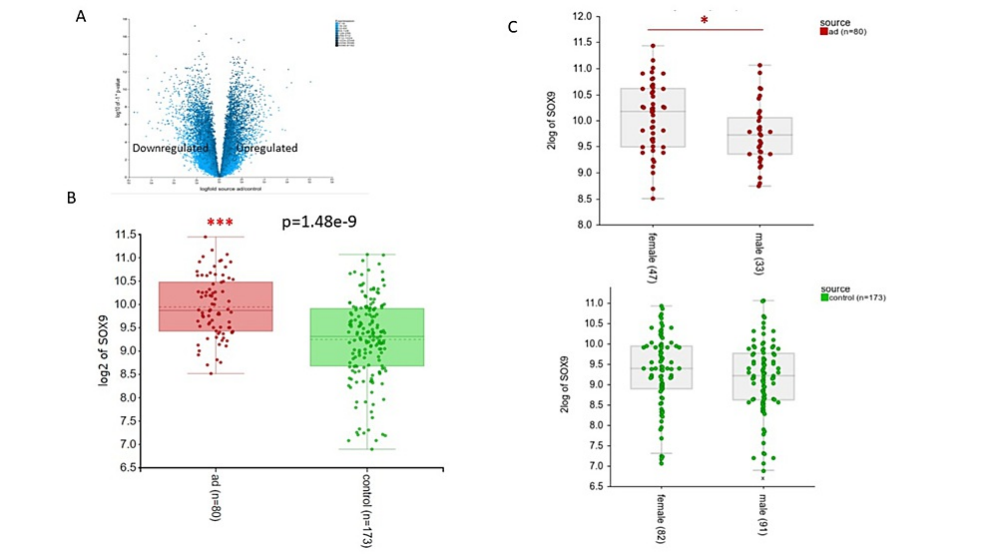
DEG and SOX9 expression in AD patients vs. control. A) Volcano plots showing the DEGs as blue dots (DEGs with p-value < 0.05) in AD patients compared to control. B) Log2 expression of SOX9 in AD cases (n=80) and control (n=173). C) Log2 expression of SOX9 in female cases vs. male cases in AD patients and control.  Data represented as median and IQR, the line in the middle of the box represents the median, the bottom line of the box represents the 25th percentile, the upper line of the box represents the 75th percentile, the lower whisker represents the minimum of the 25th percentile, the upper whisker represents the maximum of the 75th percentile. *p value < 0.05, **p < 0.01, ***p < 0.001 DEGs, differentially expressed genes; IQR, interquartile range; AD, Alzheimer's disease

A total of 7006 genes were differentially expressed (p ≤ 0.01) between AD and normal control tissues (Figure 1). Of those 3787 were upregulated in AD while the remaining 3219 were downregulated relative to control. SOX9 was significantly upregulated [p<0.001 (p=1.48e-9)] in AD tissue compared to control with a median log2 expression of 9.86 (IQR 9.42-10.47) and median of 9.31 (IQR 9.67-9.90) respectively (Figure 1). Interestingly SOX9 expression was significantly upregulated in AD female patients (p<0.05) compared to male patients. However, no significant difference in expression between genders was seen in control subjects (Figure 1).

SOX9 expression increases in the entorhinal cortex and hippocampus compared to the post-central gyrus in AD patients

**Figure 2:**
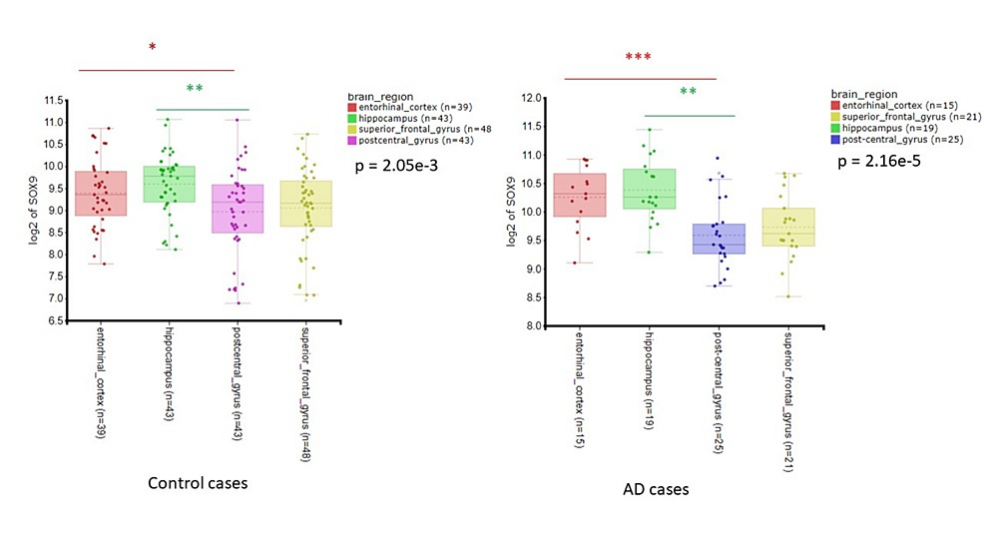
SOX9 expression in brain regions of AD patients. Log2 expression of SOX9 in four brain regions entorhinal cortex, hippocampus, post-central gyrus, and superior frontal gyrus of control cases (top graph) and AD cases (bottom graph). Data represented as median and IQR. *p < 0.05, **p < 0.01, ***p < 0.001 AD, Alzheimer's disease; IQR, interquartile range

Overall SOX9 expression was significantly different between the four brain regions whether in AD or control tissue, however, this was more prominent in AD tissue (p<0.001) compared to control tissue (p<0.01) (Figure 2). The increase in expression in AD appeared to be more in EC and HC regions compared to PCG and SFG.

SOX9 expression positively correlated with BRAAK stages

**Figure 3:**
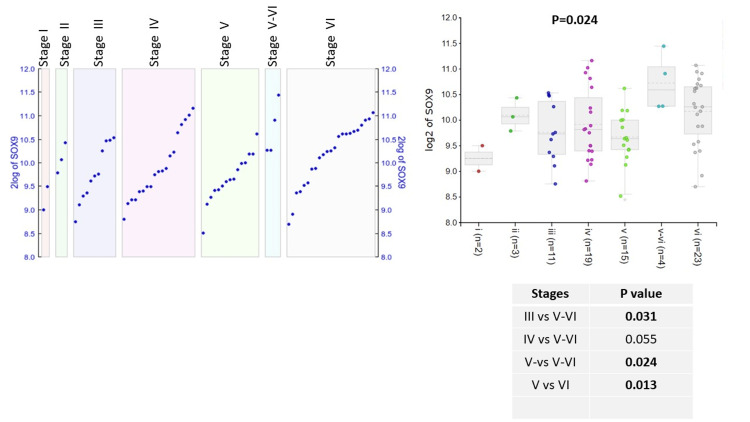
SOX9 expression in BRAAK stages of AD patients. Log2 expression of SOX9 in six BRAAK stages. Data represented as median and IQR. IQR, interquartile range

The SOX9 expression positively correlated with BRAAK stages (p<0.05) (Figure 3). Particularly stages V-VI showed a significantly higher SOX9 expression compared to stages III (p<0.05) and V (p<0.05), while expression was higher than stage IV as well this did not reach significant levels (p=0.055).

SOX9 expression is significantly lower in patients with APOE3/3 genotypes

**Figure 4:**
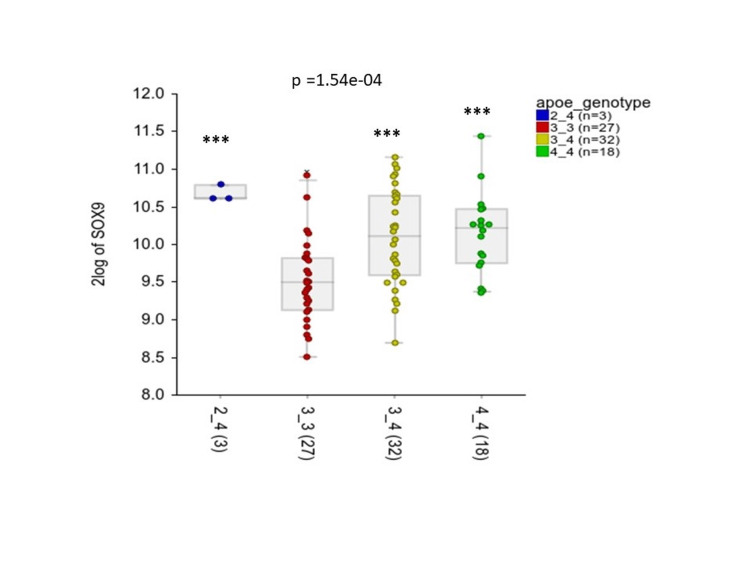
SOX9 expression in APOE genotypes of AD patients. Log2 expression of SOX9 in four APOE genotypes (APOE 2/4, APOE3/3, APOE 3/4, and APOE4/4) of AD patients. Data represented as median and IQR. ***p value < 0.001 relative to APOE3/3

The SOX9 expression significantly differed between APOE genotypes (p<0.001) (Figure 4). Overall APOE3/3 genotype showed the lowest expression with a log2 median of 9.50 (IQR 9.13-10.79) compared to APOE2/4 median 10.62 (IQR 10.65-10.79) (p<0.001), APOE 3/4 median 10.11 (IQR 9.59-11.16) (p<0.001), and APOE4/4 median 10.22 (IQR 9.75-11.44) (p<0.001) (Figure 4).

SOX9 expression negatively correlated with oxidative phosphorylation genes

Given recent reports on the metabolic regulation of SOX9, we were interested to check the correlation between SOX9 expression and oxidative phosphorylation genes expression. Interestingly SOX9 appeared to positively correlate with only 16 genes, while negatively correlate with 81 genes in the oxidative phosphorylation pathway. Mainly SOX9 expression appeared to negatively correlate with genes related to NADH dehydrogenase complex 1. Table 1 represents selected genes that negatively correlated with SOX9 expression.

**Table 1:** SOX9 expression negatively correlated with oxidative phosphorylation genes expression in brain tissue.

Gene	r	p value
ATP synthase related		
ATP5H	-0.72	3.15e-40
ATP5A1	-0.665	1.31e-32
ATP5B	-0.732	2.81e-42
ATP 5C1	-0.666	9.82e-33
ATP5L	-0.612	1.54e-26
NADH dehydrogenase [ubiquinone] 1 alpha subcomplex related		
NDUFA10	-0.707	2.54e-38
NDUFA11	-0.484	1.03e-15
NDUFA12	-0.691	4.7e-36
NDUFA13	-0.441	5.06e-13
Cytochrome c oxidase related		
COX5A	-0.755	4.03e-46
COX6B1	-0.491	3.62e-16
COX6C	-0.689	9.77e-36
Ubiquinol-cytochrome c reductase related		
UQCRQ	-0.51	1.51e-17
UQCR10	-0.508	1.92e-17
UQCRC1	-0.691	5.62e-36
UQCRC2	-0.673	1.39e-33

## Discussion

Using secondary human dataset analysis, we show for the first time that SOX9 is associated with AD and correlated with disease progression. Interestingly, in AD patients, SOX9 expression appeared more in APOE4 genotypes than APOE3, indicating a potential link between lipid metabolism and SOX9 expression in AD.

 The SOX9 is a member of a highly conserved family of TFs which contain the high mobility group DNA-binding domain. It plays a major role in organ development during embryogenesis [6]. In the brain, the TF is essential for gliogenesis switching stem cells differentiation from neurogenic to gliogenic. In SOX9 null mice neural tissue lacks astrocyte. SOX9 is not only necessary for astrocyte lineage but also for astrocyte differentiation and maintenance, and together with NFIA TF the complex activates astrocyte genes required for astrocyte maturation. Interestingly, SOX9 was found to be solely sufficient to induce an astrocyte phenotype from human pluripotent stem cell (PSC)-derived neural progenitor cells [7]. Thus an increase of SOX9 in AD tissue seen in our analysis could reflect the involvement of astrocytes in AD, and could confirm the recent interest in this cell population as a contributor for the disease that contributes significantly in the amyloid burden in the brain.

Not many studies have looked in the relationship of SOX9 with AD, however, in their recent study Xia et al. show that through targeting SOX9, miR-22-3p could inhibit apoptosis of Aβ-treated HT22 cells in vitro, in addition to improving Aβ deposition and consequently enhancing behavior in mice through the Morris water maze tests [10]. As SOX9 was concluded as the main target of miR-22-3P this study implies a role for SOX9 in AD mainly through Aβ deposition. Another study suggesting a potential link between Aβ and SOX9 showed that treating astrocytes with Aβ resulted in an increase in expression of SOX9 and Neurocan which is a proteoglycan produced largely by astrocytes [14]. Interestingly, SOX9 appeared to be responsible for the Aβ-induced Neurocan expression [14]. Overall and together with our findings these results suggest that SOX9 could be playing a role in Aβ deposition in astrocytes and also responding to Aβ deposition.

Overall, no studies have looked in the role of SOX9 in dementia, however, SOX9 expression was found to be upregulated in diseases such as multiple strokes [8]. Interestingly, a study demonstrated that SOX9 is upregulated with IBI in in vitro and in vivo models [9]. In addition, silencing of SOX9 reduced neuronal apoptosis and the inflammatory response seen with these IBI models, indicating that SOX9 can be playing a major role in promoting inflammation in IBI [9]. It would be, therefore, interesting to further examine the role of SOX9 in other brain diseases including neurodegenerative diseases.

van Gastel et al., report shows that local vasculature nutrients, specifically lipids, play an essential role in skeletal progenitor differentiation [15]. In this study, SOX9 appeared to be a key metabolic regulator which increases in response to lipid deprivation and pushes cells towards chondrogenic commitment in these conditions [15]. The SOX9 expression increased mainly in response to low oxygen and lipid deprivation, in addition SOX9 expression inhibited fatty acid oxidation. This study concludes that local nutrient might be a detrimental factor in stem cell lineage choice, and that lipid metabolism could be an essential factor for SOX9 expression and lineage effect [15]. Whether this effect also applies in astrocytes would require more studies.

 It is known that lipids are an essential component for the structure and function of the brain and play a major role whether in normal brain functions or as a contributor in disease development including AD [16]. The APOE is the major lipid carrier in the CNS and is found in three main forms APOE2,3,4 [17]. The different isoforms show different binding affinities to lipids, receptors, and Aβ; we can effect cholesterol efflux and transportation and Aβ clearance [17]. APOE4 which has a lower binding affinity for lipids than APOE2 and APOE3 is the most important risk factor for AD [18]. In human and mice studies, APOE4 allele was associated with more Aβ depositions and plaques. It is believed that APOE4 is less lipidated than other forms, which could result in more aggregation [17]. In addition astrocytes with APOE4 show less efflux of cholesterol, which could indicate that there would be a defect in cholesterol transport to neurons. Interestingly as well, APOE4 is now believed to induce a pro inflammatory response resulting in cognitive decline [19]. In our study SOX9 expression was found to be more in APOE4 genotypes compared to APOE3. Taken together one might hypothesize that lipid metabolism disruption associated with APOE4 can lead to an increase in SOX9 expression which could support astrocyte maturation and differentiation and availability in AD brain tissue, ultimately leading to disease progression. Further experiments, however, would be required to prove this.

Although there was a trend towards SOX9 expression in higher BRAAK stages, the significance was less obvious compared to its association with APOE genotypes. It is plausible that a larger number of cases would be required to prove the correlation between SOX9 and BRAAK stages. It is worth mentioning that BRAAK stages is a pathological staging which reflects deposition of a hyperphosphorylated tau protein and therefore formation of neurofibrillary tangles. The SOX9 role is more likely to be associated with Aβ depositions and metabolic regulation of astrocyte. Correlating SOX9 with clinical assessments of AD would be, therefore, useful.

Overall a main limitation of using secondary data sets is the lack of control on the data. For example, we cannot control sample size, inclusion and exclusion criteria, and the brain regions the samples were obtained from. It is also difficult to assess the quality of samples and sample preparation and overall analysis. In addition, expression and co expression profiles may not necessarily indicate a functional effect, and they do not take in consideration posttranscriptional modifications or other levels of regulations of gene expression. One way to overcome some of these problems is using multiple datasets for analysis and using supporting literature. Overall, the data provide new hypotheses that could be tested by further experiments.

## Conclusions

In conclusion, SOX9 expression is increased in AD patients specifically APOE4 genotypes. Its expression correlated negatively with oxidative phosphorylation genes, indicating a potential role of SOX9 as a metabolic regulator. The increased expression of SOX9 could be supporting astrocyte maturation, which is currently considered as an important cellular subset in the development and progression of AD. This study gives further insight for a potential role for SOX9 in AD which requires further experimentation. Studying the potential role of SOX9 as a lipid-availability sensor which in turn can alter oxidative metabolism and astrocyte function would provide more evidence for its role. Further experiments targeting SOX9 in dementia disorders can also prove whether SOX9 can be a therapeutic target in these disorders.
